# Breastfeeding practices in a public health field practice area in Sri Lanka: a survival analysis

**DOI:** 10.1186/1746-4358-2-13

**Published:** 2007-10-11

**Authors:** Suneth B Agampodi, Thilini C Agampodi, Udage Kankanamge D Piyaseeli

**Affiliations:** 1 Additional Medical Officer of Health, MOH office, Beruwala, Sri Lanka; 2 Director, National Institute of Health Sciences, Nagoda Road, Kalutara, Sri Lanka

## Abstract

**Background:**

Exclusive breastfeeding up to the completion of the sixth month of age is the national infant feeding recommendation for Sri Lanka. The objective of the present study was to collect data on exclusive breastfeeding up to six months and to describe the association between exclusive breastfeeding and selected socio-demographic factors.

**Methods:**

A clinic based cross-sectional study was conducted in the Medical Officer of Health area, Beruwala, Sri Lanka in June 2006. Mothers with infants aged 4 to 12 months, attending the 19 child welfare clinics in the area were included in the study. Infants with specific feeding problems (cleft lip and palate and primary lactose intolerance) were excluded. Cluster sampling technique was used and consecutive infants fulfilling the inclusion criteria were enrolled. A total of 219 mothers participated in the study. The statistical tests used were survival analysis (Kaplan-Meier survival curves and Cox proportional Hazard model).

**Results:**

All 219 mothers had initiated breastfeeding. The median duration of exclusive breastfeeding was four months (95% CI 3.75, 4.25). The rates of exclusive breastfeeding at 4 and 6 months were 61.6% (135/219) and 15.5% (24/155) respectively. Bivariate analysis showed that the Muslim ethnicity (p = 0.004), lower levels of parental education (p < 0.001) and being an unemployed mother (p = 0.021) were important associations of early cessation of exclusive breastfeeding. At the time of the study, 62% (135/219) of infants were receiving feeds via a bottle and 23% (51/219) were receiving infant formula. Muslim ethnicity was significantly associated with bottle and formula feeding (p < 0.001). Bottle feeding was also significantly higher among mothers with a low level of education and among employed mothers.

**Conclusion:**

The rate of breastfeeding initiation and exclusive breastfeeding up to the fourth month is very high in Medical Officer of Health area, Beruwala, Sri Lanka. However exclusive breastfeeding up to six months is still low and the prevalence of inappropriate feeding practices is high.

## Background

The single most cost effective intervention to reduce infant mortality in developing countries would be the promotion of exclusive breastfeeding. The estimated reduction of infant mortality by promoting exclusive breastfeeding is 13% [1]. Non-exclusive breastfeeding rather than exclusive breastfeeding can increase the risk of dying due to diarrhea and pneumonia among 0–5 month old infants by more than two-fold [2]. Benefits of exclusive breastfeeding up to six months duration have been studied all over the world and there are enormous amount of evidence to support this [3]. The World Health Organization recommended exclusive breastfeeding for six months in 2002 [4] and most of the international community has followed these guidelines [5,6].

Feeding a baby with mothers' milk is a well accepted and well praised behavior in the Sri Lankan culture. According to available national data, the breastfeeding initiation rate in Sri Lanka is almost 100% and 54% of mothers practice exclusive breastfeeding up to four months [7]. Data on exclusive breastfeeding up to sixth months is scarce. A study conducted in Colombo in 2003 reported that none of the study subjects were practicing exclusive breastfeeding up to sixth months [8].

The national infant feeding policy guidelines in Sri Lanka have recommended six months exclusive breastfeeding since 2005. Translation of the policy into action would need immense planning and strong implementation. In a country like Sri Lanka where the public health infrastructure is well developed compared to other countries in the region, the translation of these policies into action would seem to be easy. But we strongly believe that all public health programs originating at national level should be re-planned according to the local requirements and should be carried out as objective oriented programs in the local division.

In order to conduct a target oriented programme, it is essential to have infant feeding base-line data such as exclusive breastfeeding rates, duration of breastfeeding, and prevalence of infant formula feeding and the use of bottles. The present study aimed to collect these data in order to develop a successful programme to strengthen breastfeeding practices in the Medical Officer of Health (MOH) area of Beruwala in Sri Lanka.

## Methods

This study was a clinic-based descriptive cross-sectional study. The study was conducted in the MOH area Beruwala which is situated in the most southern part of the Kalutara district of the western province. The area is a field practice area of the National Institute of Health Sciences (NIHS). According to routinely reported data, the actual population residing in the area in 2006 was approximately 160,000. Residents of the area are comprised of multiethnic and multicultural groups. The Maternal and Child Health (MCH) care is provided by the Medical Office of Health (MOH) through Public Health Midwives and other support staff.

The study population consisted of mother-baby pairs attending Child Welfare Clinics (CWC) where the infants were between the ages of four to twelve months and currently residing in the MOH area of Beruwala. Infants with specific feeding problems (cleft lip and palate and primary lactose intolerance), thus requiring infant formula or bottle feeding, were excluded from the study as this group of infant would not be in the primary target population for the promotion of exclusive breastfeeding. The sample size was calculated assuming the rate of breastfeeding at six months of age as 20%, precision level at 0.1, confident limits 95% and with a design effect of 2. Final sample size was 211 [9]. Even though an earlier study reported six month exclusive breastfeeding as zero [8] with the policy changes and training of field and hospital staff, we estimated that it should be at least 20% by the time of the study.

A cluster sampling technique was used to collect data. A CWC clinic was considered as a cluster and there were 19 functioning clinics in the area. Clinic based cluster sampling had several advantages. The CWC clinics are used only for immunization and growth monitoring purposes. Only healthy children attend these clinics for immunization and ill children are usually referred to specialized clinics. Therefore the sample would not be "biased" as in other health facilities such as hospitals where only the "ill" children are being taken. In Beruwala area there are no private providers for immunization services and almost all infant immunizations are done in these clinics. On the other hand growth monitoring during infancy is around 95% in the area and weighing of infants less than six month of age is only done at these clinics. Therefore a highly representative sample can be achieved by conducting a clinic based study in this area.

Data were collected during June 2006 by the first two authors who had undergone lactation management master training. Consecutive infants fulfilling the inclusion criteria were enrolled in the study from each clinic until the required cluster size was reached. Study subjects were enrolled at all 19 clinics. Mothers were informed about the study and verbal consent was given during the routine infant examination. A pre-tested interviewer administered short questionnaire was used for data collection. Ethical approval was obtained from the Scientific and Ethical Review Committee, Faculty of Medicine, University of Colombo.

Data were entered into a Microsoft Access database and analyzed using SPSS 13. Percentage, proportions and contingency tables were used for description of the data. Association of inappropriate feeding practices and socio-demographic characteristics were analyzed using chi-square test. Kaplan-Meier survival analysis was used for the estimation of duration of exclusive breastfeeding because some of the infants were continuing to breastfeed exclusively (censored data) and the duration of exclusive breastfeeding was a skewed distribution. For Kaplan-Meier survival analysis, cessation of exclusive breastfeeding was taken as the final event. Individual independent variables were transformed to dichotomous variables and survival curves were compared using log-rank (Mantel-Cox) test in univariate analysis. To assess the effect of all covariates, we used the Cox proportional hazard model. Predictors of cessation of exclusive breastfeeding were evaluated using regression coefficient and Wald test.

Exclusive breastfeeding was defined according to Labbok's strict definition; that is exclusive breast feeding since birth [10]. Initiation of breastfeeding was defined as the proportion of infants receiving breast milk regardless of the time started (ever breastfed). The bottle feeding rate was defined as the proportion of infants less than 12 month of age who were receiving any food or drink through a bottle. Mothers who were actually working at the time of data collection or were employed but on maternity leave were defined as "employed".

## Results

A total of 219 infant-mother pairs were enrolled in study. The age range of infants was 4 to 12 months with median age of 6 months. Table 1 shows the characteristics of the study sample. Of the 219 mothers, 123 (56.2%) were Sinhalese and 96 (43.8%) were Muslims. Primiparous mothers accounted for 43% of the sample. Two-thirds (61.2%) of the mothers were in their twenties. Only one fourth of the mothers and one fifth of the fathers had their highest educational level beyond grade 10. Seven percent of the sample were "employed" mothers.

**Table 1:** Characteristics of the study sample (n = 219)

**Characteristics**	**n**	**%**
**Ethnicity**		
Sinhalese	123	56.2
Muslims	96	43.8

**Parity**		
Para 1	95	43.4
Para 2	75	34.2
Para 3 and above	49	22.4

**Maternal age (years)**		
<20	11	5.0
20–29	134	61.2
>30	74	33.8

**Maternal education level**		
Up to grade 10	159	72.6
Higher than grade 10	60	27.4

**Maternal Employment**		
Unemployed	203	92.7
"Employed" (includes maternity leave)	16	7.3

The breastfeeding initiation rate in this sample was 100%. Of the 219 infants, 135 (61.6%) were exclusively breastfed for four months. Only 155 infants were aged six months or more and only 24 (15.5%) of them were exclusively breastfed for six months.

The sample selected for our study was not precisely representing the ethnic composition in the Beruwala area. The proportion of Muslim mothers (43.6%) was higher than the actual proportion (32.24%) residing in the area [11]; the difference of proportions was statistically significant. This is one of the drawbacks of using the cluster sampling technique. The final exclusive breastfeeding estimation for the area could have been affected by these ethnic differences. When ethnicity-adjusted exclusive breastfeeding rates were compared using population proportions as standards, 4 months and 6 months exclusive breastfeeding rates were 63% and 19% respectively.

**Figure 1:**
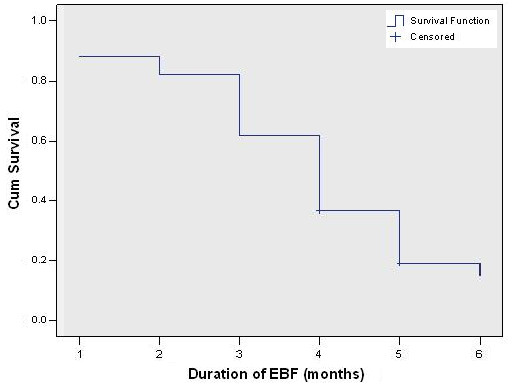
Kaplan Meier survival estimates for duration of exclusive breastfeeding.

Figure 1 shows the Kaplan-Meier survival estimates for the duration of exclusive breastfeeding. Infants who were continuing to exclusively breastfeed at the time of data collection were entered as censored data (n = 30, 13.8%). The median duration of exclusive breastfeeding was 4 months (SE 0.219, 95% Confidence Interval: 3.75, 4.25). Major drops in exclusive breastfeeding were observed after the third and fourth months.

We classified use of infant formula and feeding baby using a bottle as inappropriate feeding practices, as even expressed breast milk should be given using cup and spoon according to the national guidelines. Around two-thirds of the infants (61.6%) received feeds by bottle. The proportion of infants receiving infant formula was 23.3%.

Inappropriate feeding practices were compared with socio-demographic characteristics (Table 2). Use of bottles was significantly higher among Muslim mothers, among mothers with a lower level of education and among unemployed mothers. Even though infant formula feeding had the same pattern of association, only the ethnic differences were statistically significant (p < 0.001).

**Table 2:** Prevalence of inappropriate feeding practices according to socio-demographic characteristics

	**Bottle feeding**			**Infant formula**		
**Characteristics**	**n**	**%**	p*	**n**	**%**	p*
**Ethnicity**						
Sinhalese(n = 123)	62	50.4	**0.000**	16	13.0	**0.000**
Muslims(n = 96)	73	76.0		35	36.5	

**Maternal Education level**						
Up to grade 10 (n = 159)	105	66.0	**0.029**	41	25.8	0.154
Higher than grade 10 (n = 60)	30	50.0		10	16.7	

**Maternal Employment**						
Unemployed(n = 203)	130	64.0	**0.009**	49	24.1	0.289
Employed (16)	5	31.3		2	12.5	

* chi-square test

The use of water and other water-based foods during the first six months of life was quite high. The initial complementary feeds given to infants were water (n = 39, 17.8%), water- based food (fruit juice, kanji water, soup water etc.) (n = 19, 8.7%), infant formula (n = 26, 11.9%). Of the mothers who started early complementary feeding before six months, only 47 (21.5%) had started food in semi-solid form.

**Figure 2:**
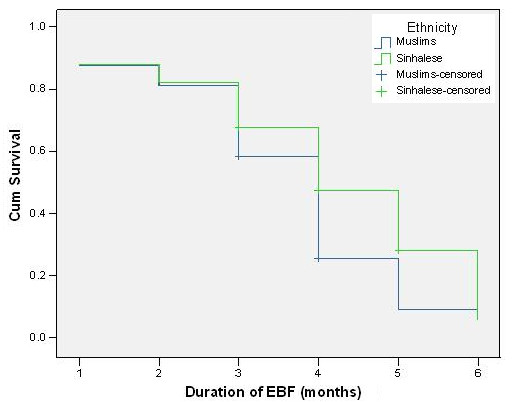
Comparison of ethnic groups regarding survival data on exclusive breastfeeding.

**Figure 3:**
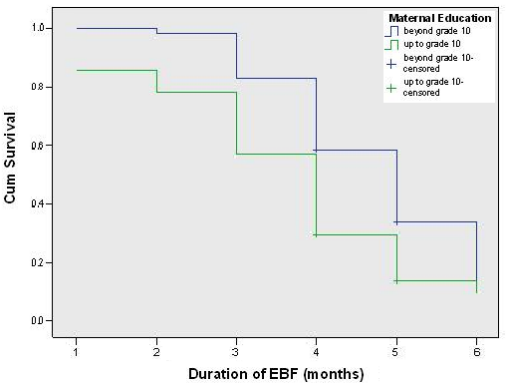
Comparison of maternal educational level regarding survival data on exclusive breastfeeding.

Comparison of survival curves indicated that only ethnicity, parental education and maternal employment were significantly associated with the duration of exclusive breastfeeding. During the first two months exclusive breastfeeding survival curves of both ethnic groups was similar (Figure 2). After the second month, the exclusive breastfeeding rate had dropped among Muslim mothers more rapidly compared to Sinhalese mothers (log-rank test: chi square = 8.34, p = 0.004). The exclusive breastfeeding survival curves of both mothers (Figure 3) and fathers (not shown) with higher level of education were constantly higher than those of parents with lower level of education. Comparison of survival curves using the log-rank test indicated highly significant differences, for both mothers (p < 0.001, chi square = 16.217) and fathers (p < 0.001, chi square = 17.084).

**Figure 4:**
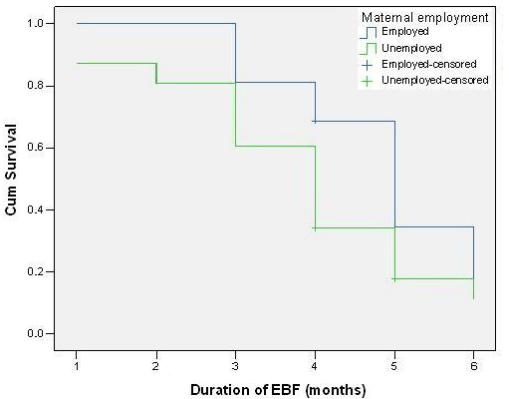
Comparison of maternal employment status regarding survival data of exclusive breastfeeding.

The exclusive breastfeeding survival curve of "employed" mothers was constantly higher than that of non-employed mothers (Figure 4). Around 70% of "employed" mothers continued exclusive breastfeeding until completion of fifth month, whereas only 60% of "non employed" mothers continued exclusive breastfeeding up to four months.

All independent variables that were significantly associated with exclusive breastfeeding in univariate analysis were entered into the Cox proportional hazard model to evaluate the covariates on hazard function for cessation of exclusive breastfeeding and to remove the effect of confounders. In this model, ethnicity and maternal employment was entered as categorical variables, while other variables were entered as interval data. None of these variables were significant predictors of cessation of exclusive breastfeeding in this multivariate model. Table 3 summarizes the results of the Cox regression analysis.

**Table 3:** Regression coefficient of covariates on cessation of exclusive breastfeeding

	**Regression coefficient**	**Wald test**	**Significance (p)**
Birth order	-0071	0.449	0.503
Age of the mother	0013	0.371	0.542
Paternal education	-0051	1.191	0.275
Maternal education	-0020	0.146	0.702
Non-working mother	0343	0.990	0.320
Sinhalese ethnicity	-0277	2.277	0.131

## Discussion

Breastfeeding practices in Sri Lanka are considered the best in South East Asia [12].

Over the past decade, reported exclusive breastfeeding practices were increasing in the country. There has been number of studies on breastfeeding in Sri Lanka [8,13-16] but published data on six month exclusive breastfeeding are scarce.

In countries where lactation support is available, six months exclusive breastfeeding has improved substantially over the time [4]. Estimated current exclusive breastfeeding rate for six months duration is 58% according to the Sri Lanka report card on breastfeeding published by the International Baby Food Action Network (IBFAN) [10]. This is a fairly reasonable estimation based on the 54% of four months exclusive breastfeeding rate in the year 2000. National data for six months exclusive breastfeeding is yet to be published. The estimate reported in our study is well below expectations. This may be due to several reasons. A major determinant of exclusive breastfeeding practices has been the health care providers' knowledge, attitudes and skills for promotion of exclusive breastfeeding. A knowledge assessment survey done on exclusive breastfeeding in 2006, assessing all public health midwives (n = 70) in the NIHS field practice area reported that only 50% of midwives had a good understanding on the existing policy on recommendation of six months exclusive breastfeeding [17]. It seems that the policy recommendation of "four to six months" exclusive breastfeeding, which was practiced for several years, had not been well understood by most of the health care providers. Change of recommendation from "four to six months" to six months has not penetrated to the grass root level public health staff. This may be the reason that the four months exclusive breastfeeding rate is much higher than six months exclusive breastfeeding rate in this area. When we consider four months exclusive breastfeeding rate, it was better than the national average and could be one of the highest documented rates for Sri Lanka.

A decline in exclusive breastfeeding after the fourth month is common elsewhere in the world [4]. Still we believed this drastic decline from 63% to 19% is highly unsatisfactory for a field practice area of the NIHS, as prerequisites such as social support and proper health care infrastructure with adequate resources to implement six months exclusive breastfeeding had been already available in the area.

Prevalence of suboptimal feeding practices among infants was very high among the study participants. The bottle feeding rate reported in the present study (61.6%) was much higher than the study by Jayathilaka and Fernando in 2002, which was 44% [16]. This difference cannot be attributed to ethnic differences because Sinhalese infants also had a 50% bottle feeding rate. This will be another major area where we should focus on when planning infant feeding programs.

Both bottle feeding and formula feeding practices were significantly higher among the Muslim ethnic group. This finding should be analyzed very carefully. Language barrier has been a problem for provision of public health care provision in Sri Lanka [18]. Lack of Tamil speaking midwives may have contributed to this finding. But, cultural and religious practices and practices among local subgroups affect exclusive breastfeeding rates all over the world. Studies in various countries have reported significant differences of exclusive breastfeeding rates among ethnic groups.

Factors affecting breastfeeding have being reviewed extensively [15,19-23]. Maternal education and women's employment were two major determinants described by most of those reviews as being associated with exclusive breastfeeding. In the present study, parental education was significantly associated with exclusive breastfeeding and it also showed a positive correlation of exclusive breastfeeding with maternal employment. This may simply be because of small number of employed mothers in the sample. However, it may be a reflection of maternity benefits received by working mothers through the extended maternity leave provision commenced in 2006. (Government provides 84 days fully paid maternity leave followed by optional 84 days half paid maternity leave and 84 days no pay maternity leave). Nevertheless, we recommend further studies to investigate the relationship between maternal employment and exclusive breastfeeding in Sri Lanka.

There were several limitations in our study. It was conducted as a base-line data collection before the implementation of an exclusive breastfeeding promotion programme. Therefore the sample size calculation was done using 0.1 as precision level which resulted in a small sample size which could have affected some of our conclusions. There was a probability of overestimation of proportion of exclusive breastfeeding due to selection bias. In Sri Lanka infants who attend CWC clinics regularly are more likely to receive better health education, support and motivation to continue exclusive breastfeeding. Therefore clinic based sampling procedure may systematically overestimate the final proportion. Another limitation of our study was the use of Muslim mothers who were fluent in Sinhalese as translators. This may have introduced biased estimation of exclusive breastfeeding rates, most probably an overestimation. As the data collectors were also service providers for the area there was a probability of underreporting of unacceptable feeding practices and overestimation of exclusive breastfeeding. Nevertheless the study found low exclusive breastfeeding rate and high proportion of unacceptable feeding practices.

## Conclusion

Our study revealed that the recommendation of six months exclusive breastfeeding is not properly implemented in the MOH area of Beruwala. This might indicate deficiencies of policy implementation at field level which could be equally applied to other parts of the country. Proper planning and results based programs through the existing system is needed with close monitoring and timely evaluation to transform policy recommendations to action at a field level. These results need urgent attention of programme planners as well as divisional level service providers.

## Competing interests

The author(s) declare that they have no competing interests.

## Authors' contributions

SBA participated in the design, data collection, and manuscript preparation and performed the data analysis. TCA participated in the design, data collection and manuscript preparation. UKDP participated in design and helped to draft the manuscript. All authors read and approved the final manuscript.

## References

[B1] Jones G, Steketee RW, Black RE, Bhutta ZA, Morris SS (2003). How many child deaths can we prevent this year?. Lancet.

[B2] Arifeen S, Black RE, Antelman G, Baqui A, Caulfield L, Becker S (2001). Exclusive breastfeeding reduces acute respiratory infection and diarrhea deaths among infants in Dhaka slums. Pediatrics.

[B3] World Health Organization (2002). Report of the expert consultation on the optimal duration of exclusive breastfeeding.

[B4] World Health Organization (2002). Global Strategy on Infant and Young Child Feeding. 55th World Health Assembly.

[B5] National Health and Medical Research Council (2003). Dietary Guidelines for Children and Adolescents in Australia incorporating the Infant Feeding Guidelines for Health Workers.

[B6] American Academy of Pediatrics. Policy statement (2005). Breastfeeding and the use of human milk. Pediatrics.

[B7] Department of Census and Statistics in collaboration with Ministry of health nutrition and welfare 2002 (2000). Sri Lanka Demographic and Health Survey.

[B8] Bundusena ASL (2003). Selected determinants and sequelae of exclusive breastfeeding up to six month in infants attending hospital and field well baby clinics.

[B9] Lwanga SK, Lemeshow S (1991). Sample size determination in health studies A practical manual.

[B10] Labbok M, Krasovec K (1990). Toward consistency in breastfeeding definitions. Studies in Family Planning.

[B11] DCS online Number and percentages of population by district. Census 2001.

[B12] International Baby Food Action Network (2006). The state of the world's breastfeeding, Sri Lankan report card.

[B13] Senanayake MP, Weerawarna H, Karunaratne KW, De Silva TU (1999). Do babies need water in Sri Lanka?. Ceylon Med J.

[B14] Sorensen E, Fernando DN, Hettiarachchi I, Durongdej S, Podhipak A, Skaara BB (1998). Exclusive breastfeeding among women on the plantations in Sri Lanka. J Trop Pediatr.

[B15] Jayasuriya D, Sosa P (1974). Feeding studies in Ceylonese babies. J Trop Pediatr Environ Child Health.

[B16] Jayathilaka CA, Fernando DN (2002). A community-based study on breastfeeding practices in Gampaha district. Journal of the College of Community Physicians of Sri Lanka.

[B17] Agampodi SB (2007). Annual Action Plan 2007, MOH area-Beruwala.

[B18] Agampodi SB (2006). Utilization of private sector for immunization of children in Colombo municipal council area.

[B19] Forman MR (1984). Review of research on the factors associated with choice and duration of infant feeding in less-developed countries. Pediatrics.

[B20] Koktürk T, Zetterström R (1989). The promotion of breastfeeding and maternal attitudes. Acta Paediatr Scand.

[B21] Popkin BM, Bilsborrow RE, Akin SA, Yamamoto ME (1983). Breast-feeding determinants in low-income countries. Med Anthropol.

[B22] Simopoulos AP, Grave GD (1984). Factors associated with the choice and duration of infant-feeding practice. Pediatrics.

[B23] Wilmoth TA, Elder JP (1995). An assessment of research on breastfeeding promotion strategies in developing countries. Soc Sci Med.

